# A machine learning algorithm to improve building performance modeling during design

**DOI:** 10.1016/j.mex.2019.10.037

**Published:** 2019-11-02

**Authors:** Chanachok Chokwitthaya, Yimin Zhu, Robert Dibiano, Supratik Mukhopadhyay

**Affiliations:** a Department of Construction Management, Louisiana State University, Baton Rouge 70803, USA; b Ailectric LLC., 7117 Florida Blvd, Baton Rouge 70806, USA; c Department of Electrical Engineering and Computer Science, Louisiana State University, Baton Rouge 70803, USA

**Keywords:** A framework for combining context-aware design-specific data and building performance models to improve building performance predictions during design, Building performance models, Contextual factors, Occupant behaviors, Immersive virtual environments, Artificial neural network, Feature ranking

## Abstract

Building design involves the optimization of factors affecting building performance such as building functions, comfort, safety, and energy. Building performance models (BPMs) help designers to evaluate and optimize such factors. However, the lack of design capabilities to validly describe human-building interactions for buildings under design may contribute to the development of inaccurate BPMs and the performance discrepancy between predictions and actual buildings. To address this challenge, a computational framework is proposed to increase the estimations performance of BPMs. The framework uses artificial neural networks (ANNs) to combine *an existing BPM* and *context-aware design-specific data* describing design-specific human-building interactions captured by using immersive virtual environments (IVEs). The framework produces an augmented BPM that can predict building performance taking human-building interactions specific to a new design into consideration. It incorporates a feature ranking technique allowing designers to assess impacts of contextual factors on human-building interactions. The paper focuses on providing details of theories, experiment and data collection designs, and algorithms behind the framework as a companion paper of [1].

• A framework for combining contextual factors with building performance models to enhance their predictive performance.
• Computation for determining impacts of contextual factors on human-building interaction.

## Graphical abstract

**Figure:**
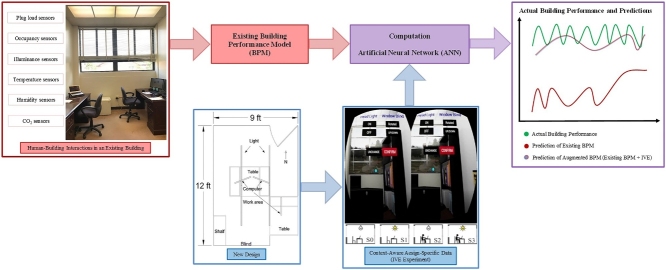

**Specifications Table**

**Table:** 

Subject Area:	Engineering
More specific subject area:	Building performance models (BPMs) IVE for studying human-building interactions
Method name:	A framework for combining context-aware design-specific data and building performance models to improve building performance predictions during design
Name and reference of original method:	[14] C. M. Bishop, *Neural networks for pattern recognition*. 1995 [10] S. Saeidi, C. Chokwitthaya, Y. Zhu, and M. Sun, “Spatial-temporal event-driven modeling for occupant behavior studies using immersive virtual environments,” Autom. Constr., vol. 94, no. May, pp. 371–382, 2018.
Resource availability:	The framework is evaluated by using two main data sources. [8] D. R. G. Hunt, “Predicting Artificial Lighting Use- A Method Based Upon Obseved Patterns of Behavior,” Light. Res. Technol., vol. 12, no. 1, pp. 7–14, 1980. The paper provides an existing building performance model. [10] S. Saeidi, C. Chokwitthaya, Y. Zhu, and M. Sun, “Spatial-temporal event-driven modeling for occupant behavior studies using immersive virtual environments,” Autom. Constr., vol. 94, no. May, pp. 371–382, 2018. The paper provides the procedure to design experiment in an immersive virtual environment (IVE) and collect context-aware design-specific data.

## Overview

During design, designers widely use building performance models (BPMs) to analyze, understand, and predict building systems, energy usages, occupancy comfort, safety, and health. BPMs are usually constructed based on data of human-building interactions obtained using traditional data collection methods (e.g., surveys, sensors, and laboratories). These methods are heavily reliant on existing buildings. Consequently, data of human-building interactions collected in such a manner does not effectively describe those interactions in new designs. This often contributes to the discrepancy between estimations and real building performance, which has often been cited as a major impediment towards the achievement of building performance objectives [[2], [3], [4]].

To that end, the authors have offered a computational framework to reduce the abovementioned discrepancy by improving the prediction accuracy of BPMs. The framework enhances the prediction accuracy of existing BPMs by incorporating *context-aware design-specific data* associated with new designs, which allows designers to finetune existing BPMs using the context information in new designs. Immersive virtual environments (IVEs) are used to simulate building contexts of building under design as well as observe and collect human-building interactions. Artificial neural networks (ANNs) combine *an existing BPM* with *context-aware design-specific data* acquired by using IVEs.

The paper focuses on details of theories, algorithms, experimental designs, and data collections of the framework. Full research and validations of the framework can be found in [1].

## Method details

### Computational framework

**Fig. 1:**
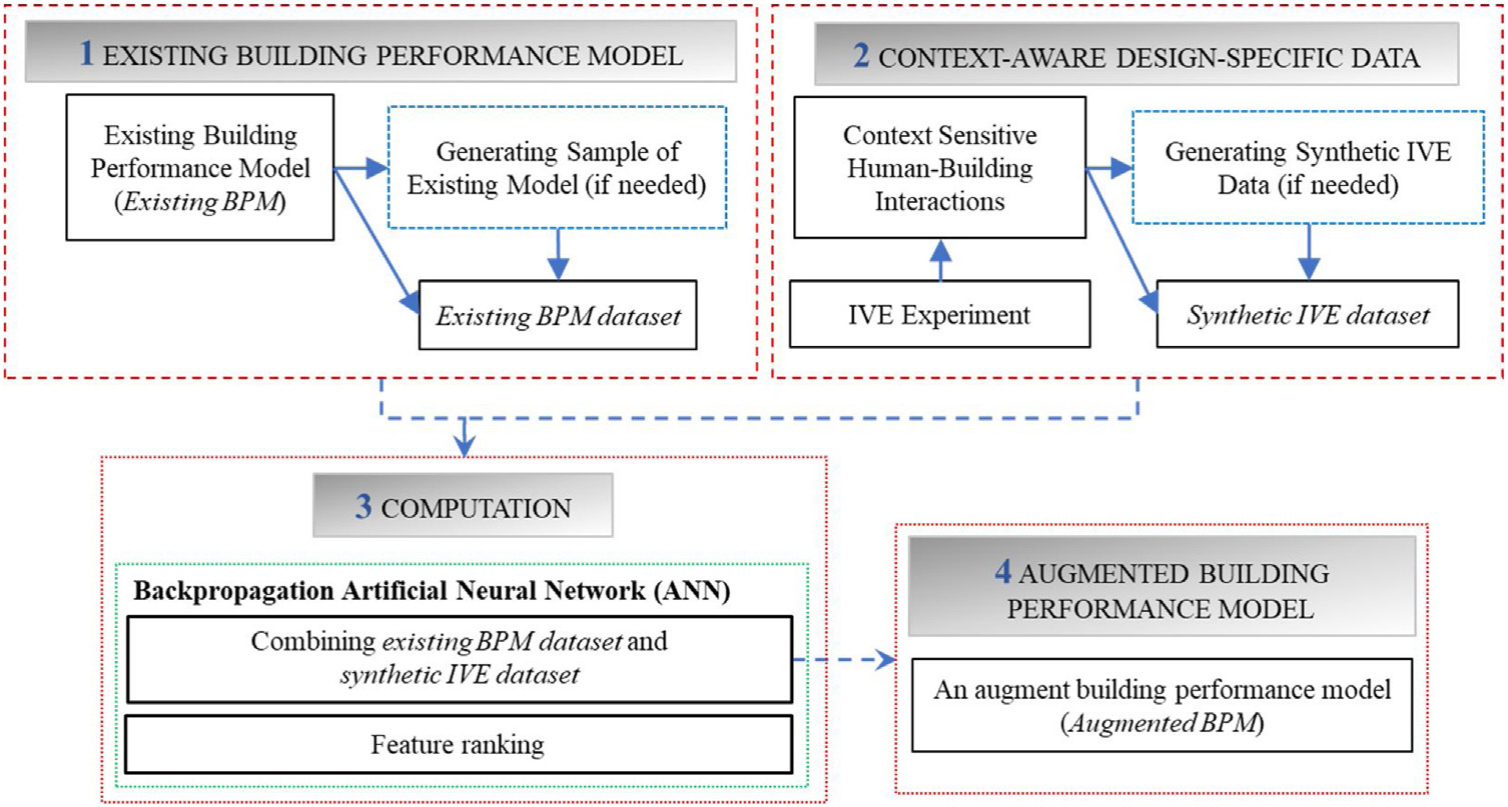
The computational framework [1].

There four main elements included in the computational framework (see Fig. 1); (1) an existing building performance model (*an existing BPM*), (2) *context-aware design-specific data* obtained from IVE experiments, (3) computation, and (4) *an augmented BPM*. In the following, theories, algorithms, experimental designs, and data collections of the components are elaborated in detail.

### Existing building performance model

An existing building performance model (*an existing BPM*) presents relationships between dependent variables such as human-building interactions and independent variables such as interior configurations, locations of building components, and outdoor environments. *Existing BPMs* can be in a variety of forms such as statistical models (e.g., regression) and occupancy data [[5], [6], [7]].

**Fig. 2:**
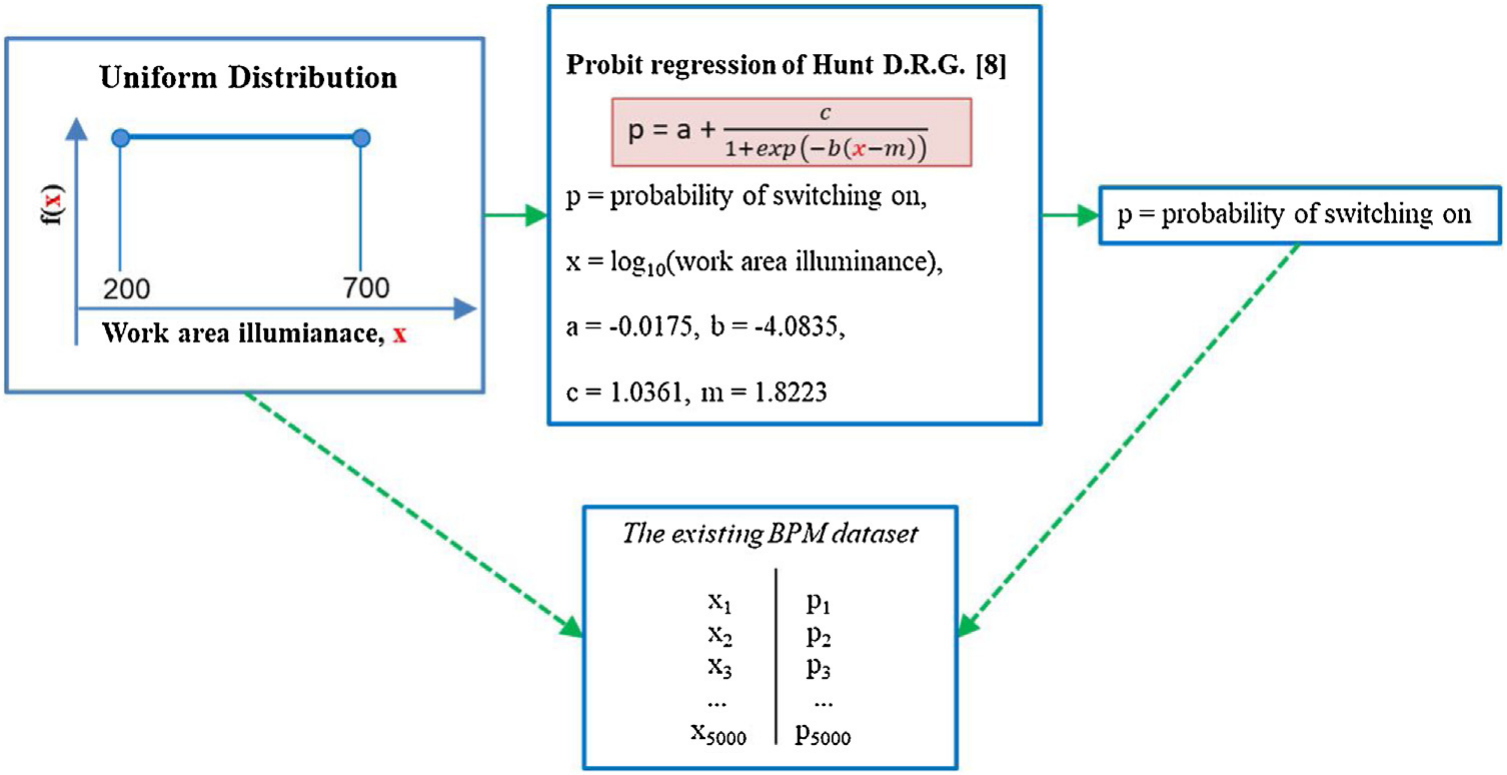
The diagram of executing the existing building performance model.

To demonstrate the framework, the authors chose a lighting BPM developed by Hunt [8] as *an existing BPM.* The Hunt model is in the form of Probit regression (see Fig. 2). Monte Carlo (MC) simulation is applied to acquire independent and identically distributed (IID) samples of *the existing BPM*. In the MC simulation, work area illuminance (*x*) is considered as inputs. A uniform distribution is used to randomly generated work area illuminance with range from 200 lux to 700 lux. The uniform distribution is used because values of the work area illuminance are assumed to occur with the same relative frequency. The MC simulation used work area illuminance (lux) and Hunt model to produce the probabilities of switching on. The obtained IID samples of work area illuminance (lux) and the corresponding probability of switching on are paired, called *the existing BPM dataset*, and comprised of 5000 data points. The number of data points are determined based on the learning curve approach [9]. The learning curve is a plot between the number of training data and the accuracy of the trained ANN with a specific number of iterations. Up to a certain point, additional training data do not significantly increase the accuracy of the trained ANN (called knee point). The number of training data point is defined based on the knee point. Details of ANNs are explained in the computation section.

### Context-aware design-specific data

**Fig. 3:**
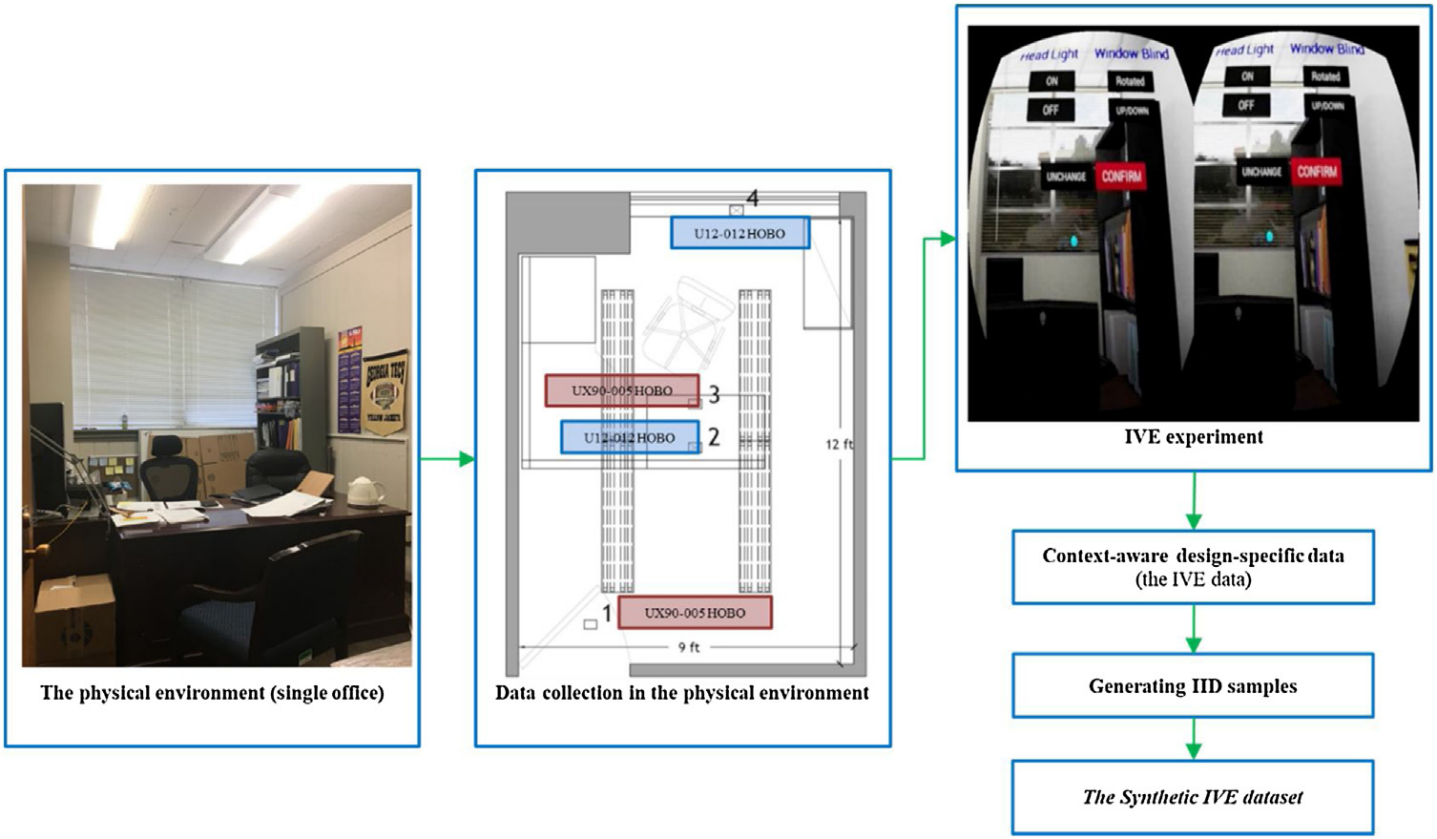
Diagram of *context-aware design-specific data* [1].

Fig. 3 illustrates steps to obtain *context-aware design-specific data* (IVE data) and how to synthetically generate IID samples from obtained IVE data (*the synthetic IVE dataset*). The details of each step are explained in the following sections.

#### The physical environment

An office is selected as the physical environment (see Fig. 3). The dimension is 9’ x 12’ x 10’ (width × length × height). The office is equipped with multiple sensors to measure the following: 1) indoor and outdoor illuminance (lux), 2) the light switch status (on and off), and 3) the occupancy statuses (occupy and non-occupy) as described in Table 1. The sensors collect data with 5 s intervals between September 23rd and October 27th, 2016.

**Table 1:** Descriptions and locations of the sensors installed in the office.

Sensor	Measurement	Location	# in Fig. 3
Onset UX90-005 HOBO occupancy/light	The occupancy and the lighting status	Above the entrance door	1
Onset UX90-005 HOBO occupancy/light	The occupancy and the lighting status	On the work area (desk)	3
Onset U12-012 HOBO temperature/relative humidity/light/ data loggers	The work area illuminance	On the work area (desk)	2
Onset U12-012 HOBO temperature/relative humidity/light/ data loggers	The outdoor illuminance	On the window	4

The data of the occupancy obtained from the physical environment are observed with respect to occupant interactions’ patterns with the light switch. Contextual information of factors influencing interactions are also investigated and defined (e.g., occupancy status, length of intermediate leaving, outdoor illuminance, and work area illuminance). Factors influencing human-building interactions on light switch usages are summarized as shown in Table 2 and they are used to develop the IVE experiments. Moreover, the data obtained from the physical environment are used as a baseline to evaluate results of *an augmented BPM*.

**Table 2:** Contextual factors, independent, and dependent variables in the case study.

Contextual Factor (*Observation*)	Status
Occupancy	Non-occupy (False)
	Occupy (True)
Intermediate Leaving	No-leave
	Short intermediate leave (shorter than an hour)
	Long intermediate leave (longer than or equal to an hour)
Outdoor Illuminance	Dark
	Normal
	Bright
**Independent Variable (*Observation*)**	**Status**
Work Area Illuminance	Dark (200 Lux)
	Normal (500 Lux)
	Bright (700 Lux)
**Dependent Variable (*State*)**	**Status**
Light Switch	On (S_1_)
	Off (S_2_)

#### IVE experiment

The IVE experiment is designed by using the Spatial-Temporal Event-Driven (STED) modeling approach [10] along with the occupancy data obtained from the physical environment. Based on the STED, the IVE experiment is constructed by using four major variables, i.e., states, contexts, events, and human(H)-building(B) interactions. States are the statuses of operations in the building at the certain point of time, i.e., light on and off in the IVE experiment (see Table 2). Contexts are situations of independent variables and the contextual factors in Table 2, which describe conditions of the building at the certain point of time. Events are occurences such as events during a day (arrival, intermediate leaving, and departure) that set contexts as well as influence the occupant interacions changing or maintaining the state. H-B interactions refer to occupant interactions with building components (e.g., light switch), which are triggered by the occurances of events.

**Fig. 4:**
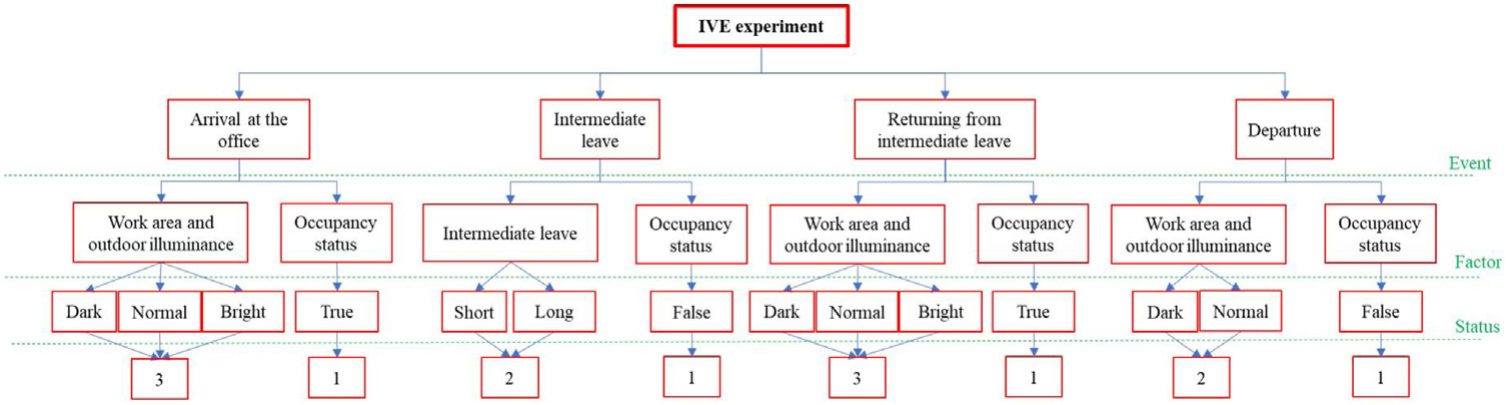
Diagram of factors included in the IVE experiment [1].

IVE experiments are arranged in sequences. Each sequence is comprised of multiple events, namely initial, arrival, intermediate leaving, coming back from intermediate leaving, and departure (see Table 3). The unique combination of factors in the IVE experiment are defined by events described in Fig. 4. Three events of the arrival, two events of the intermediate leave, three events of the returning from intermediate leave, and two events of the departure lead to 3 × 2  ×  3 × 2 = 36 sequences. For example, the first sequence represents: (1) at arrival, bright illuminance and occupy, (2) at intermediate leave, long intermediate leave and non-occupy, (3) at returning from intermediate leave, bright illuminance and occupy, and (4) at departure, normal illuminance and non-occupy.

**Table 3:** The sequence of the IVE experiment.

Event	Sequence of IVE Experiment in a Sequence			
	*Light Status Before Interaction*	*Virtual and Auditory Cues Exposed to the Participant*	*Interaction*	*Light Status After Interaction*
**Arrival at the Office**	Initial light status		Participant interacts with light switch	Light status of the event
**Intermediate Leave**	Light status of the previous event		Participant interacts with light switch	Light status of the event
**Returning from the Intermediate Leave**	Light status of the previous event		Participant interacts with light switch	Light status of the event
**Departure**	Light status of the previous event		Participant interacts with light switch	Light status of the event

In the IVE experiment, visual and auditory cues are exposed to the participant to inform the participant about the situations of variables in Table 2. The prticipant is a male faculty member in Louisiana State University, who also occupies the physical environment. During the IVE experiment, the roll of the participant is to select the light switch status based on given events. There are three alternative light switch statuses for the participant to select, namely switch on, off, and maintain the light switch. The IVE experiment occurs in two sessions and lasts 140 min in total. Occupancy status, work area illuminance, outdoor illuminace, and intermediate leaving status data as well as the selections of the light switch status are recorded throughout the experiment. The recorded data are called *context-aware design-specific data*.

#### Generating IID samples from IVE experiment

Since the sample size of *context-aware design-specific data* is relatively small, IID samples are generated by using a Hidden Markov Model (HMM) Baum-Welch [11]. The HMM learns the relationship between factors influencing human-building interactions (i.e., occupancy, intermediate leaving, work area and outdoor illuminance) and human-building interactions (i.e., light switching). The HMM assumes that, in each sequence, the current state at time *t* (*S_t_*) influences occurrence of the adjacent state at time *t + 1* (*S_t+1_*). The state changes from the current state at time *t* to the next state at time *t + 1* is describe as a state transition [12]. The time steps of data collected from the IVE experiment are presented in Table 4. For instance, if the light is on (*S_t_*) at the arrival event (*t*), the situation of the light on may influence the occupant to turn off the light or leave the light on (*S_t+1_*) at the intermediate leaving event (*t + 1*). The probabilities of state transitions are analyzed. A transition probability matrix is used to present the probabilities of state transitions. The observations are sets of contexts occurring under particular events. For example, at the arrival event (*t*), the observation is occupied office, no leave, dark outdoor and work area illuminance. The probabilities of observations are calculated and simplified in an observation probability matrix.

**Table 4:** Time steps of data collected from the IVE experiment.

	Time step	
	t	t+1
**Event**	Initial	Arrival at the Office
	Arrival at the Office	Intermediate Leave
	Intermediate Leave	Returning from the Intermediate Leave
	Returning from the Intermediate Leave	Departure

The IVE experiment data are classified into the states and the observations of events (see Table 2). The states are the statuses of the light switch. The other variables are observations. Each observation is encoded in a vector form and represented as an ordinal variable. The example of an encoded observations is described as follow: occupancy, no intermediate leave, bright work area illuminance, and bright outdoor illuminance are represented as “*Occupy + No leave + Bright + Bright*”. Then, it is represented by “*1*”. After that, the initial-state, transition probabilities and observation probabilities are analyzed.

Initial-state probabilities are probabilities that states (*s_n_*) in Table 2 occur at initial events in 36 sequences (*p(s_n_)*), which can be calculated using (1).

(1) $$\text{p}({\text{s}}_{\text{n}})\;=\frac{Number\;of\;times\;the\;S_{n}\;occurs\;in\;initial\;events}{total\;number\;of\;intial\;events}$$

In this study, the initial light status is randomly assigned with light on and off equally likely throughout the 36 sequences. Therefore, the initial-state probabilities are 0.5 for both light switch on (S_1_) and light switch off (S_2_).

Transition probabilities are probabilities of state changes from event e (*S_i_*) to event e+1 (*S_j_*) across the experiment (*p(S_i_, S_j_)*). The formula to obtain transition probabilities is shown in (2).

(2) $$\text{p}({\text{S}}_{\text{i}},\;{\text{S}}_{\text{j}})\;=\frac{Number\;of\;occurences\;that\;S_{i}\;at\;event\;e\;changes\;to\;S_{j}\;at\;event\;e+1\;}{Total\;number\;of\;occurences\;of\;S_{i}\;}$$

Transition probabilities of this study are calculated and demonstrated in Table 5, where S_1_ is light switch on and S_2_ is light switch off.

**Table 5:** Transition probability matrix of this application.

		Transition probability	
		**S_t+1_ occurred at e+1**	
		*S_1_*	*S_2_*
**St occurred at e**	*S_1_*	0.35	0.65
	*S_2_*	0.96	0.04

Observation probabilities are probabilities that an observation occurs under each state. The formula to obtain observation probabilities is shown in (3). The observation probability matrix of this study is obtained and shown in Table 6.

(3) $$\text{p}({\text{S}}_{\text{i}},\;\text{K})\;=\frac{Number\;of\;occurences\;of\;observation\;K\;{associated\;with\;S}_{i}}{Total\;number\;of\;occurences\;of\;S_{i}\;}$$

**Table 6:** Observation probability matrix of this application.

Status of observations	Observation probability											
	Non-occupy + No leave + Dark + Dark	Non-occupy + No leave + Normal + Normal	Non-occupy + No leave + Bright + Bright	Non-occupy + Short leave + Dark + Dark	Non-occupy + Short leave + Normal + Normal	Non-occupy + Short leave + Bright + Bright	Non-occupy + Long leave + Dark + Dark	Non-occupy + Long leave + Normal + Normal	Non-occupy + Long leave + Bright + Bright	Occupy + No leave + Dark + Dark	Occupy + No leave + Normal + Normal	Occupy + No leave + Bright + Bright
State1 (*S_1_*)	0.06	0.06	0.06	0.04	0	0.04	0	0	0	0.25	0.25	0.24
State2 (*S_2_*)	0.29	0.29	0.07	0.03	0.07	0.03	0.07	0.07	0.07	0	0	0.01

**Fig. 5:**
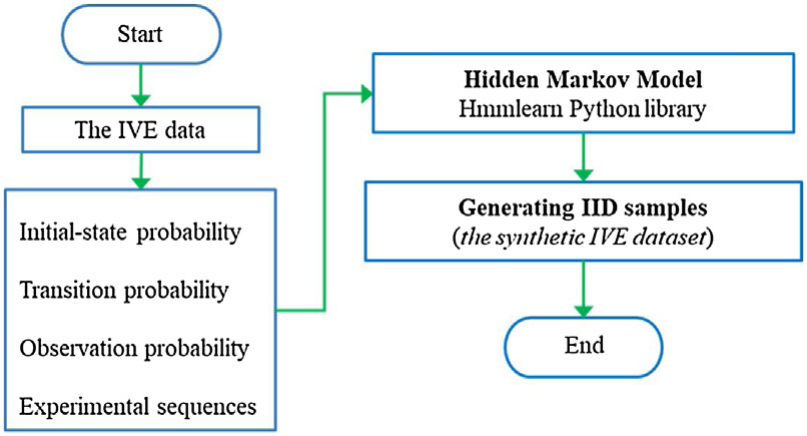
IID samples of the IVE data.

Then the HMM takes the initial-state, the transition and the observation probabilities calculated previously, and the 36 sequences of observations as inputs in the training process. Fig. 5 shows IID samples from the IVE experiment data, where the HMMLearn Python library is used for the training and application of the HMM [13].

The trained HMM produces 5000 data points of the statuses of the light switch, the independent variables, and the contextual factors. Like *the existing BPM dataset*, the number of data points are determined by using the learning curve approach [9]. The probabilities of switching on are analyzed by using data of statuses of the light switch. Then, the probabilities of switching on, the IID samples of the independent variables, and the contextual factors are paired. The paired dataset is called *the synthetic IVE dataset*.

### Computation

**Fig. 6:**
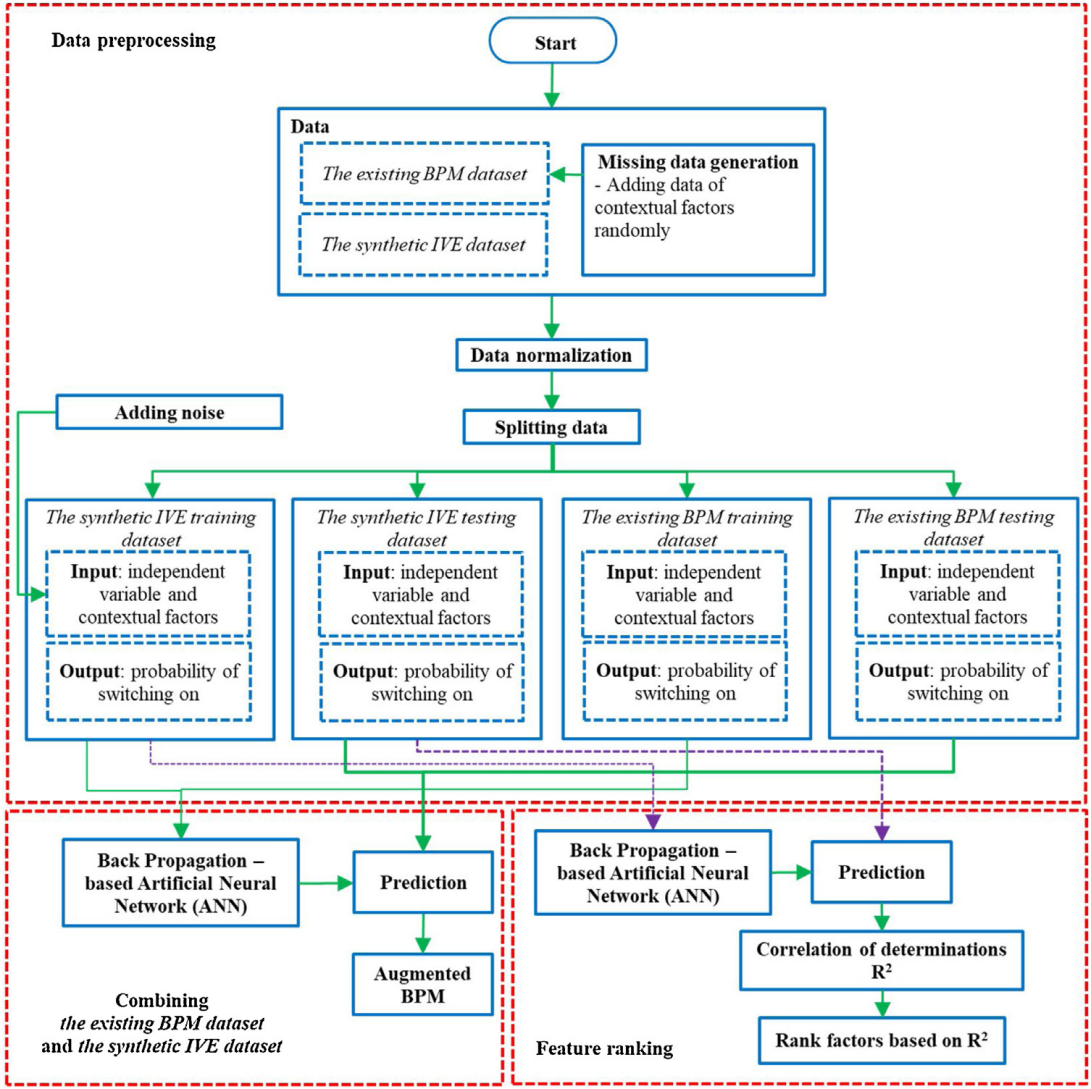
Flowchart representing the computation of the framework.

The core of the framework is the computation for biasing *an existing BPM dataset* by using *a synthetic IVE dataset* to enhance the performance of *the existing BPM*. Fig. 6 demonstrates the major stages of the computation in the framework (i.e., data pre-processing, combination of *the existing BPM dataset* and *the synthetic IVE dataset*, as well as feature ranking), which are explained in the following.

#### Data pre-processing

Four major data preprocessing steps are performed, namely missing data generation, data normalization, data splitting, and adding Additive White Gaussian Noise (AWGN).

**Fig. 7:**
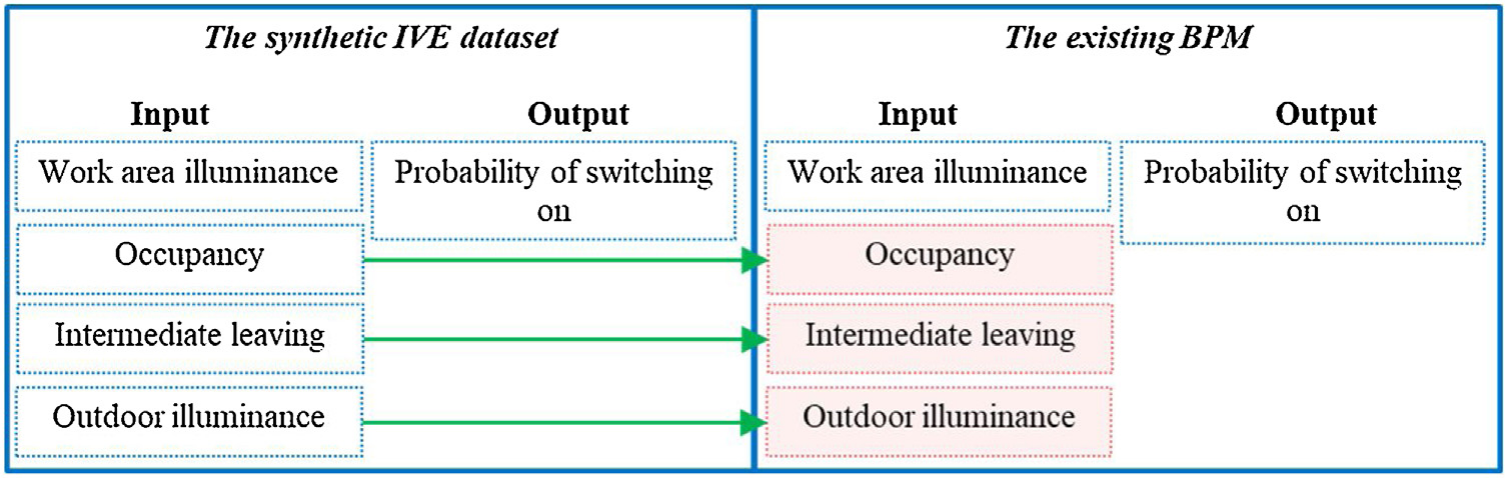
Diagram of generating missing data for *the existing BPM dataset*.

**Missing data generation:** Since *the existing BPM dataset* does not include contextual factors, data of contextual factors are randomly generated by replicating contextual factors in *the synthetic IVE dataset* (see Fig. 7). The descriptions are as follows:

• The data of occupancy are generated by using variables of non-occupancy and occupancy.
• The data of intermediate leaving are generated by using variables of non-leave, short intermediate leave, and long intermediate leave.
• The data of outdoor illuminance are generated by using variables of dark, normal, and, bright.

**Data normalization:** The input data (i.e., an independent variable and contextual factors) of *the existing BPM dataset* and *the synthetic IVE dataset* are normalized with respect to the standard deviations and means of *the synthetic IVE dataset*. The probabilities of switching on (outputs) of both datasets are not normalized.

**Data splitting:** The normalized *existing BPM dataset* and *synthetic IVE dataset* are separated based on an 80-20 split as follows:

1) training datasets, which include:
  a *the existing BPM training dataset*
  b *the synthetic IVE training dataset*
2) testing datasets, which include:
  a *the existing BPM testing dataset,*
  b *the synthetic IVE testing dataset*

**Fig. 8:**
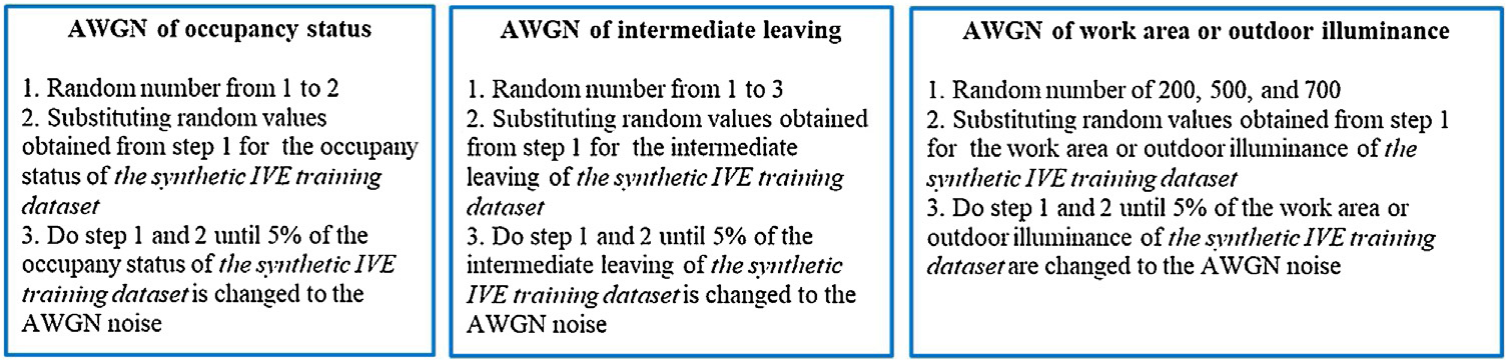
Steps to calculate the AWGN for *the synthetic IVE training dataset*.

**Adding noise:** five percent of *the synthetic IVE training dataset* is substituted for *Additive white Gaussian noise (AWGN)* to increase the variability of the data and reduce overfitting during the computation process. The steps of generating AWGN for *the synthetic IVE training dataset* are explained in Fig. 8.

#### Combining the existing BPM dataset and the synthetic IVE dataset

**Fig. 9:**
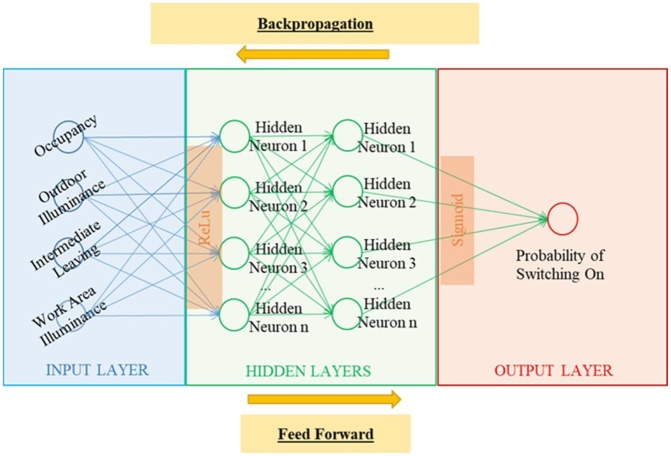
Scheme of the Back Propagation – based Artificial Neural Network (ANN) [1].

**Back Propagation-based Artificial Neural Network (ANN)**: The framework combines *the existing BPM dataset* and *the synthetic IVE dataset* by using the Back Propagation-based Artificial Neural Network (ANN) [14]. The computational process is constructed by using the Python language. The ANN system is built based on the Keras functional application program interface (API) [15]. The three-layered ANN comprises of the input, hiddens, and output layers (see Fig. 9). The input layer involves the data of the following: 1) occupancy, 2) outdoor illuminance, 3) work area illuminance, and 4) intermediate leaving from mixtures of *the existing BPM training dataset* and *the synthetic IVE training dataset*. The output layer takes the data of the probability of switching on from mixtures of both training datasets. The hidden layers use 300 hidden neurons per layer with rectified linear unit activation function (ReLU). The output layer uses sigmoid activation function. The regularization is elastic net regularization (combination of L1 (Laplacian) and L2 (Gaussian) penalties). The loss function uses binary cross entropy (logistic regression). The regularization and learning rate are 10^−6^.

**Fig. 10:**
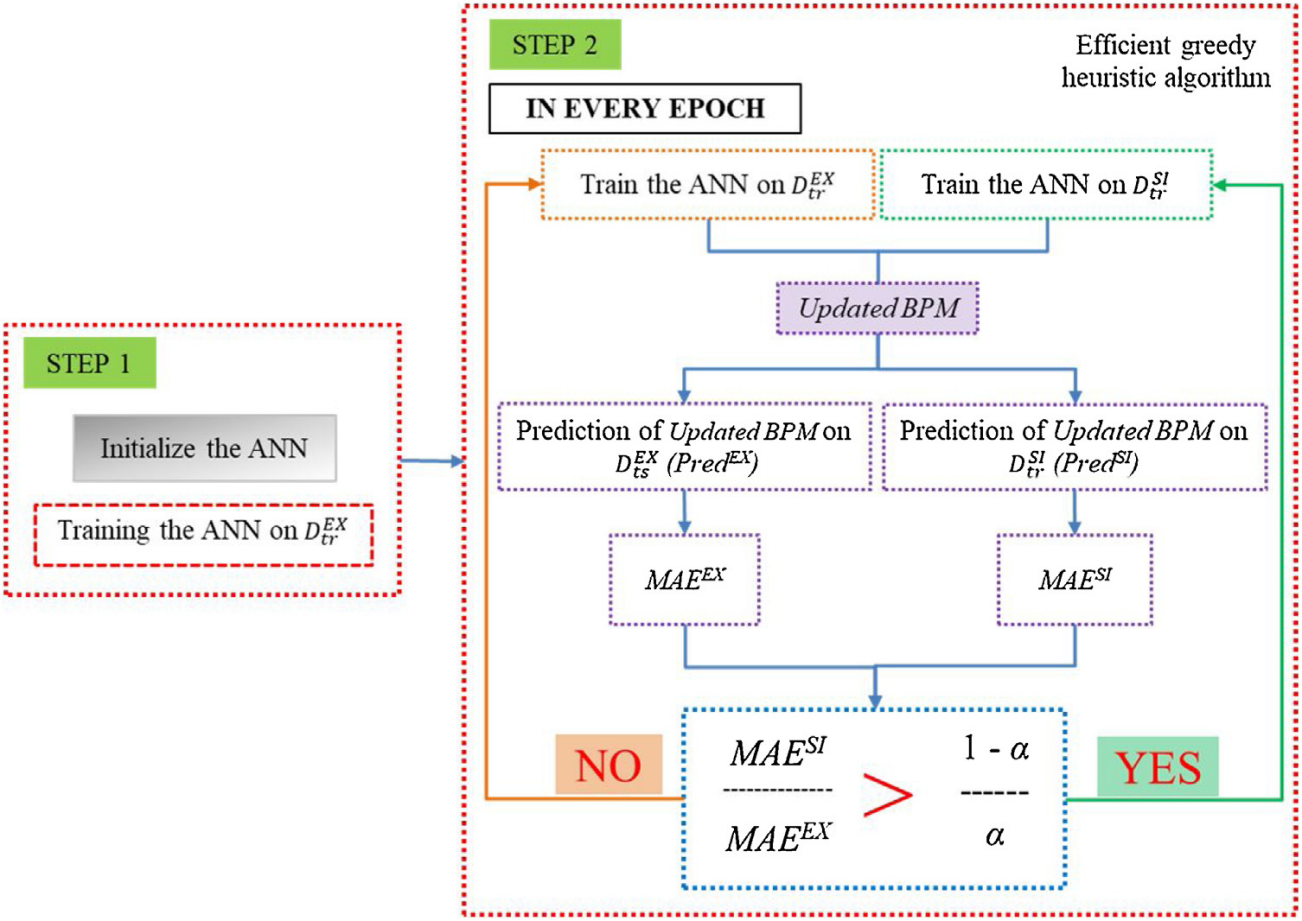
Training algorithm.

**Training Algorithm:** The ANN is trained by using the algorithm shown in Fig. 10, where notations are described in Table 7. The ANN is initialized by training it with *the existing BPM training dataset* ($D_{tr}^{EX}$) for 60,000 epochs (see step 1 in Fig. 10). After initialization, the ANN is trained on *the existing BPM training dataset* ($D_{tr}^{EX}$) and *the synthetic IVE training dataset* ($D_{tr}^{SI}$) for various mixture ratios by following step 2 described in Fig. 10. A mixture ratio (*α*), a number between 0 and 1, is defined to determine a mixture of *the existing BPM dataset* ($D_{tr}^{EX}$) and *the synthetic IVE dataset* ($D_{tr}^{SI}$). The ANN is trained by using an efficient greedy heuristic algorithm. The mean absolute errors (MAEs) are used as measurements to specify whether the ANN should be trained on *the existing BPM training dataset* or *the synthetic IVE training dataset* in each epoch. The MAE is used in the algorithm in two aspects. First, the MAE^EX^ measures errors between the expected outputs of *the existing BPM testing dataset* ($O_{ts}^{EX}$) and the predictions of an updated BPM on *the existing BPM testing dataset* (*Pred^EX^*), which can be calculate by using Eq. (4). Second, the MAE^SI^ measures errors between the expected outputs of *the synthetic IVE testing dataset* ($O_{ts}^{SI}$) and the predictions of an updated BPM on *the synthetic IVE testing dataset* (*Pred^SI^*), which can be calculate by using Eq. (5). The notations used in Eqs. (4) and (5) are described in Table 7.

(4) $${\text{M}\text{A}\text{E}}^{\text{E}\text{X}}=\frac{\sum_{1}^{\text{N}\text{EX}}\left|O_{ts}^{EX}-\text{Pred}\text{EX}\right|}{\text{N}\text{EX}}$$

(5) $${\text{M}\text{A}\text{E}}^{\text{S}\text{I}}=\frac{\sum_{1}^{\text{N}\text{SI}}\left|O_{ts}^{SI}-\text{Pred}\text{SI}\right|}{\text{N}\text{SI}}$$

**Table 7:** Notations of variables used in the training algorithm (Fig. 10).

Variables	Notation
*The existing BPM training dataset*	$D_{tr}^{EX}$
*The synthetic IVE training dataset*	$D_{tr}^{SI}$
*The existing BPM testing dataset*	$D_{ts}^{EX}$
*The synthetic IVE testing dataset*	$D_{ts}^{SI}$
*The expected outputs of*$D_{ts}^{EX}$	$O_{ts}^{EX}$
*The expected outputs of*$D_{ts}^{SI}$	$O_{ts}^{SI}$
The number of data points in $D_{ts}^{EX}$	*N_EX_*
The number of data points in $D_{ts}^{SI}$	*N_SI_*
Prediction of the ANN on $D_{ts}^{EX}$	*Pred^EX^*
Prediction of the ANN on $D_{ts}^{SI}$	*Pred^SI^*

At every epoch, if $\frac{{MAE}^{SI}}{{MAE}^{EX}}$ > $\frac{1-\text{α}}{\text{α}}$, the algorithm greedily attempts to reduce $\frac{{MAE}^{SI}}{{MAE}^{EX}}$ in this epoch. This is done by training *the updated BPM* on *the synthetic IVE training dataset* in this epoch that reduces the *MAE^SI^* and increases the *MAE^EX^*. Otherwise, in that epoch, *the updated BPM* is trained on *the existing BPM training dataset*, i.e., $\frac{{MAE}^{SI}}{{MAE}^{EX}}$ increases. In this study, the process continues for 400,000 epochs. The 400,000 epochs are defined based on many trials of the number of epochs started from 50,000 with an interval of 50,000 epochs. The learning curve [9] approach is used to investigate the errors of the predicted outcomes of the learned ANN by plotting the values of the MAEs (i.e., *MAE^SI^* and *MAE^EX^*) and the number of epochs. The values of the MAEs remain almost the same, when the number of epochs is higher than 400,000. Therefore, the 400,000 epochs are used throughout the study.

Several combinations of *the existing BPM dataset* and *the synthetic IVE dataset* are constructed based on given mixture ratios (α). The obtained results of combinations are “*updated BPMs*”. Each *updated BPMs* is evaluated against the data from the physical building. The *updated BPM* that has the least errors when evaluated against the data from the physical building becomes *an augmented BPM*.

#### Feature ranking

**Fig. 11:**
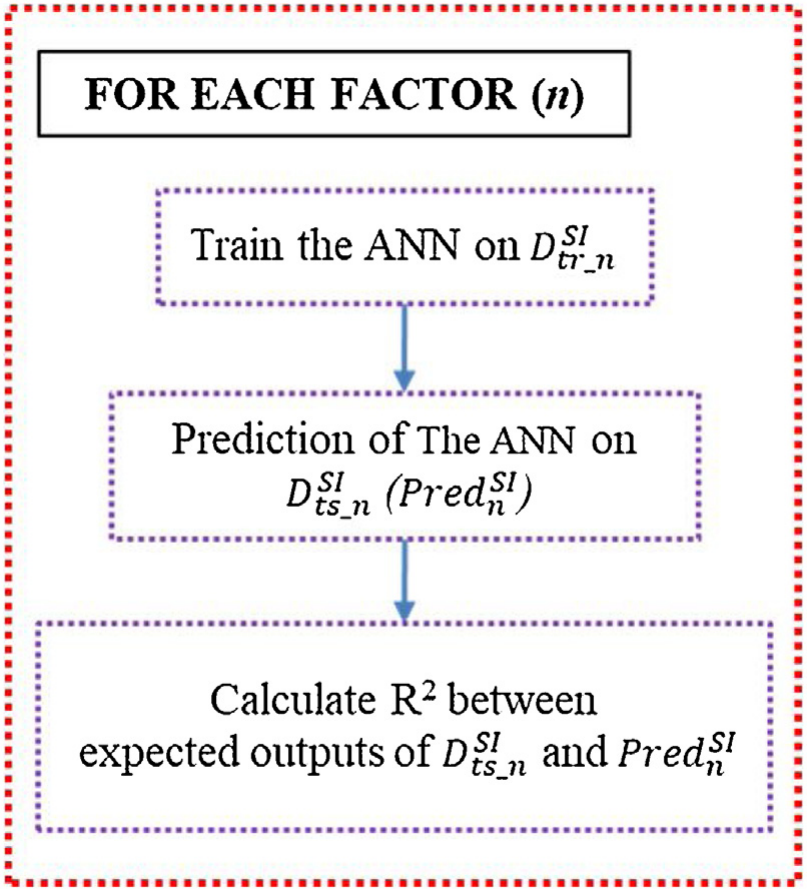
Feature ranking algorithm.

In this study, factors impacting predictions include: 1) occupancy status, 2) intermediate leaving, 3) work area illuminance, and 4) outdoor illuminance, which their levels of impacts are certainly different. Feature ranking determines the relative impact of such factors. The feature ranking uses three-layered ANN similar to Fig. 9 for evaluating the level of impact of each factor. To evaluate the impact of each factor, *the synthetic IVE training dataset* and *the synthetic IVE testing dataset* are modified so that the input to the ANN contains only one factor of interest a time. For example, evaluating the impact of occupancy status on the prediction of probability of switching on can be performed by having only data of occupancy status as the input to the ANN and the output remains the same (i.e., the probability of switch on). The ANN is trained by the modified *synthetic IVE training dataset* for 400,000 epochs. Then, the ANN predicts the outputs on *the modified synthetic IVE testing dataset*. The correlation of determinations (R^2^) statistically indicate how accurate the learning of the ANN by calculating linear relationships between the expected outputs and predicted outputs [16]. R^2^ can be in range from 0 to 1. If R^2^ is close to or equal to 1, the predictions of the ANN have low or without errors, meaning a factor strongly impact on the prediction of the ANN. The algorithm of the feature ranking and notations are demonstrated in Fig. 11 and Table 8 respectively.

**Table 8:** Variables and Notations used in the feature ranking algorithm (Fig. 11).

Variables	Notation
*The synthetic IVE training dataset with only factor of interest (n) as input*	$D_{tr\_n}^{SI}$
*The synthetic IVE testing dataset with only factor of interest (n) as input*	$D_{ts\_n}^{SI}$
Prediction of the ANN on $D_{ts\_n}^{SI}$	${Pred}_{n}^{SI}$

### Limitation and future work

Several potentials have been demonstrated through the application of the framework [1]. However, limitations of the framework still exist with respect to the following aspects:

• The framework requires users to define mixture ratio manually. The optimal mixture may be difficult to obtain since users may not accurately approximate the mixture in advance. To enhance the effectiveness of the framework, a different approach is needed to determine an optimal mixture without using a trial-and-error method, for example using the energy efficiency goal of a building to determine the mixture ratio [17,18].
• The results of the study are obtained from one participant, which may affect the observational data significantly. More cases and the variety of participants need to be considered in future studies.
• The numbers of iterations to train the ANN in this application are defined by using a pre-specified number of epochs, which must be high enough to ensure the proper training and accurate outcomes. As a result, computational resources (e.g., time, memory space, and storage) may be excessively consumed. In the future work, an algorithm will be developed to determine the convergence point for training the ANN, which may reduce the number of epochs and the use of computational resources. For instance, an algorithm determines the differences of the mean absolute error (MAE) between a previous and a current epoch (early stopping). If the MAE of the current epoch is less than the MAE of the current epoch for a specific number (user defined number), the training is converged.

### Conclusions

The paper elaborates the technical details (e.g., theories, experimental and data collection designs, and algorithms) behind the computational framework discussed in [1]. The main purpose of the framework is to increase the estimation performance of BPMs. The framework combines *an existing BPM* with *context-aware design-specific data* by using the ANN and produce *an augmented BPM*. *An augmented BPM* has better estimations of human-building interactions than *an existing BPM*. Human-building interactions are captured using immersive virtual environments (IVEs). Moreover, the framework provides designers or researchers the feature ranking technique to investigate the impact of contextual factors.

The framework involves the application of different methods, e.g., existing BPMs, *context-aware design-specific data*, ANNs, and feature ranking. It is validated using an existing BPM retrieved from [8], *context-aware design-specific data* retrieved from [10], and occupancy data retrieved from a physical environment. The validation of the framework is presented in [1].
